# Contribution of RdDM to the ecotype-specific differential methylation on conserved as well as highly variable regions between Arabidopsis ecotypes

**DOI:** 10.1186/s12864-023-09128-4

**Published:** 2023-01-20

**Authors:** Jaehoon Lee, Sang-Yoon Shin, Sang-Kyu Lee, Kyunghyuk Park, Haechan Gill, Youbong Hyun, Choongwon Jeong, Jong-Seong Jeon, Chanseok Shin, Yeonhee Choi

**Affiliations:** 1 Department of Biological Sciences, Seoul National University, Seoul, 08826 South Korea; 2 Research Center for Plant Plasticity, Seoul National University, Seoul, 08826 Republic of Korea; 3 Interdisciplinary Program in Agricultural Genomics, Seoul National University, Seoul, 08826 Republic of Korea; 4 Graduate School of Green-Bio Science and Crop Biotech Institute, Kyung Hee University, Yongin, 17104 South Korea; 5 Current address: Division of Life Science, Gyeongsang National University, Jinju, 52828 South Korea; 6 Department of Agricultural Biotechnology, Seoul National University, Seoul, 08826 Republic of Korea; 7 Research Institute of Agriculture and Life Sciences, Seoul National University, Seoul, 08826 Republic of Korea; 8 Plant Genomics and Breeding Institute, Seoul National University, Seoul, 08826 South Korea

**Keywords:** *Arabidopsis*, Methylome, Transposable elements (TEs), Structural variation, RNA-directed DNA methylation (RdDM)

## Abstract

**Background:**

Several studies showed genome-wide DNA methylation during *Arabidopsis* embryogenesis and germination. Although it has been known that the change of DNA methylation mainly occurs at CHH context mediated by small RNA-directed DNA methylation pathway during seed ripening and germination, the causality of the methylation difference exhibited in natural *Arabidopsis* ecotypes has not been thoroughly studied.

**Results:**

In this study we compared DNA methylation difference using comparative pairwise multi-omics dynamics in Columbia-0 (Col) and Cape Verde Island (Cvi) ecotypes. *Arabidopsis* genome was divided into two regions, common regions in both ecotypes and Col-specific regions, depending on the reads mapping of whole genome bisulfite sequencing libraries from both ecotypes. Ecotype comparison was conducted within common regions and the levels of DNA methylation on common regions and Col-specific regions were also compared. we confirmed transcriptome were relatively dynamic in stage-wise whereas the DNA methylome and small RNAome were more ecotype-dependent. While the global CG methylation remains steady during maturation and germination, we found genic CG methylation differs the most between the two accessions. We also found that ecotype-specific differentially methylated regions (eDMR) are positively correlated with ecotype-specifically expressed 24-nt small RNA clusters. In addition, we discovered that Col-specific regions enriched with transposable elements (TEs) and structural variants that tend to become hypermethylated, and TEs in Col-specific regions were longer in size, more pericentromeric, and more hypermethylated than those in the common regions. Through the analysis of RdDM machinery mutants, we confirmed methylation on Col-specific region as well as on eDMRs in common region are contributed by RdDM pathway. Lastly, we demonstrated that highly variable sequences between ecotypes (HOT regions) were also affected by RdDM-mediated regulation.

**Conclusions:**

Through ecotype comparison, we revealed differences and similarities of their transcriptome, methylome and small RNAome both in global and local regions. We validated the contribution of RdDM causing differential methylation of common regions. Hypermethylated ecotype-specific regions contributed by RNA-directed DNA methylation pathway largely depend on the presence of TEs and copy-gain structural variations. These ecotype-specific regions are frequently associated with HOT regions, providing evolutionary insights into the epigenome dynamics within a species.

**Supplementary Information:**

The online version contains supplementary material available at 10.1186/s12864-023-09128-4.

## Background

DNA methylation is an epigenetic modification at the 5′ position of a cytosine base, altering DNA accessibility and chromatin structure in eukaryotes. DNA methylation is known to regulate gene expression and gene imprinting, as well as transposon silencing and genome integrity [1]. While cytosine methylation occurs mainly at CG symmetric sites in mammals, plants exhibit a more comprehensive system; methylation occurs not only at CG sites but also at CHG and CHH sites (H = A, T, or C) [2, 3]. In *Arabidopsis thaliana*, a model flowering plant, only about 14% of genes contain CG methylation (mCG) exclusively in the transcribed gene body, whereas heterochromatic TE and repeat sequences are highly methylated at all three sites [4].

The de novo methylation at all three cytosine contexts is mediated by small RNA-directed DNA methylation (RdDM) pathway. Then, symmetric methylated CG (mCG) is maintained during DNA replication by DNA METHYLTRANSFERASE 1 (MET1) [5]. Symmetric methylated CHG (mCHG) is maintained by CHROMOMETHYLASE 3 (CMT3) using its chromodomain that recognizes H3K9 methylation [6, 7]. Asymmetric CHH methylation (mCHH) is only maintained by de novo methylation due to the lack of mCHH on the parental DNA strand [1, 8, 9]. Heterochromatic regions, including TE or repeat elements, are transcribed by plant-specific RNA polymerase IV (pol IV), and the transcripts are processed into 24-nucleotide(nt) small RNAs or 21-nt aberrant small RNAs. These small RNAs act together with pol V-derived transcripts and recruit the de novo methyltransferase, DOMAIN REARRANGED METHYLTRANSFERASE 2 (DRM2), to methylate its target regions at all three cytosine contexts. Also, chromodomain-containing CMT2 methylates CHH and, to a lesser extent, CHG, mainly at pericentromeric heterochromatic regions independently of small RNAs [10, 11].

Since 24-nt small RNAs were found to play essential roles in plant methylome regulation, many studies exist investigating their mechanisms [12–14] and their impacts on plant biology [15, 16]. In recent years, there have been considerable advances in genomic difference-linked diversity in methylome between *Arabidopsis* ecotypes and their involvement in evolution and adaptation [17–19]. However, these studies have not dissected the epigenetic roles of small RNAs in natural variations. Studies profiled the small RNA dynamics of developing and germinating seeds, which provided valuable information on small RNA dynamics with epigenomic insights [20–23]; however, the interplay between methylome and small RNAome linked to natural variation in *Arabidopsis* is not well understood. Although the global change of DNA methylation during seed maturation and germination has been previously reported for *Arabidopsis* and soybean, the observations are limited to a single reference ecotype, Col [24, 25], or from different ecotypes, such as Ws-0 and Col, that are used during embryogenesis and germination, respectively [26]. Therefore, a pairwise comparison of DNA methylation differences accompanying association analyses for the small RNA and mRNA expressions is required to understand the mechanisms underlying epigenetic differences found in natural accessions.

Here, we present a genome-wide comparison of the DNA methylome, small RNAome, and transcriptome dynamics during seed ripening and germination in Col and Cvi. We classified *Arabidopsis* genome into two regions based on the mapping characteristic of whole-genome bisulfite sequencing (WGBS) reads: comparable common regions (CR), where the sequence reads from both Col and Cvi are well mapped; Col-specific regions (Col SR), where only the Col WGBS reads are well mapped (See Methods and materials section for the detailed conditions). We demonstrate that ecotype-specific differentially methylated regions (eDMRs) determined in CR strongly correlated with highly expressed 24sRC (24-nt small RNA Cluster) in an ecotype-specific manner. We also discovered significantly higher methylation levels in the Col SR than CR with accompanying 24sRC, in an ecotype-specific and RdDM-dependent manner. Then, we present several factors responsible for higher methylation levels in Col SR, providing evolutionary insight into how different methylation levels in different ecotypes originated from natural variation within a species.

## Results

### CG ecotype-specific differentially methylated regions (eDMRs) are strongly maintained in the gene body throughout development

To understand variation in the molecular dynamics during seed ripening and germination processes, we performed NGS-based multi-omic analysis, including the transcriptome, small RNAome, and DNA methylome, on seeds sampled from two *Arabidopsis* accessions, Col and Cvi. We chose Cvi to compare to the reference, Col, since Cvi is known to have the lowest CG gene body methylation (gbM) among over 1000 *Arabidopsis* accessions [19]. Seeds were sampled from three stages: freshly harvested (FH) fully matured green seeds from green plants, after-ripening (AR) seeds harvested from fully dried plants, and germination-stimulated seeds (GS) (see Method, Additional file 1: Fig. S1).

For DNA methylome analysis, we used fractional window without overlap. The level of methylation was calculated by dividing the number of mC with the number of mC plus T at single base. The methylation levels included in a window were averaged for representing the methylation level of a window. The size of window varied from each analysis (see Methods in detail).

**Fig. 1:**
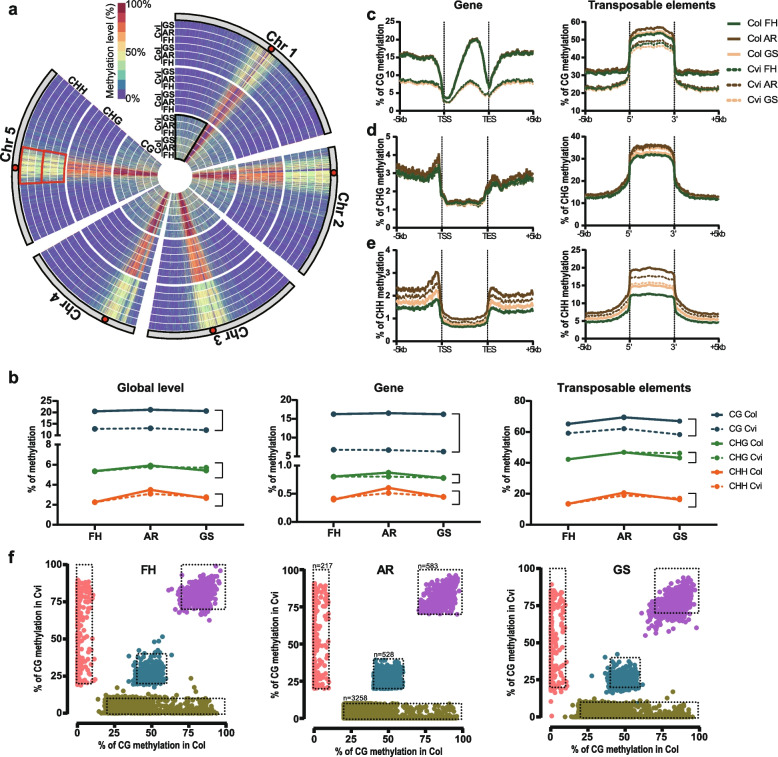
DNA methylation during seed ripening and germination in both ecotypes. **a** Chromosomal DNA methylation view. Red-colored circle indicates centromere. Sections in black or red indicate each pericentromere and chromosomal arm, respectively. **b** Average of DNA methylation level (%) of Col (lined) and Cvi (dotted) was calculated according to developmental stages in global (left panel), gene (middle panel), and transposable elements (right panel). **c** Stable difference of genebody methylation level between Col and Cvi. Genes including more than 5 sites of analyzed cytosine context with at least 10 reads were analyzed. Four differentially colored sections were selected at the AR stage for tracing the change in other stages. Sectioned several parts from the AR stage, methylated in Col but not Cvi (copper metallic), methylated in Cvi but not Col (pink), middle level in both ecotypes (azure blue), and highly methylated in both ecotypes (purple). **(d–f)** The methylation level of gene, transposable elements, and surrounding regions were analyzed for each cytosine context

For a global methylome view, we used comparable common regions where were mapped to TAIR10 in both ecotypes using our WGBS libraries (see Methods). Figure 1a shows the chromosomal heat map of mCG, mCHG, and mCHH during development. The most striking difference between the two ecotypes is observed in mCG at chromosomal arms (e.g. black sector), both ecotypes show a distinctly higher mCHH change at pericentromeric regions (e.g. red sector) in the AR stage (Fig. 1a).

mCG did not globally change during the three developmental stages, maintaining the methylation difference between the two ecotypes (Fig. 1b). In contrast, mCHG and mCHH were the highest in the AR stage, while their methylation levels were almost the same in the two ecotypes (Fig. 1b). The dynamics of the genebody methylation (gbM) mirrored the global view (Fig. 1b, c-e). The non-CG methylation of TE showed the similar dynamic pattern to the non-CG genic regions, except for the higher methylation levels (Fig. 1b, d, e). On the other hand, the mCG pattern of TE was different from the gene or global view; AR seeds exhibited the highest methylation levels (Fig. 1b, c). These results suggest that Cvi, which is known to have the lowest mCG levels among all ecotypes [19], display the same mCHH dynamics during seeds maturation and germination as Col, at least for their conserved regions [24–26]. Therefore, cytosine methylation tends to reach maximal levels at the AR stage and then decrease during germination, although their levels in each accession are different. Since the levels of non-CG methylation were very similar in both ecotypes and mCG were significantly different, the mCG difference is the primary source of the total methylation difference between Col and Cvi.

Since the global mCG level ecotype differences, but not mCHG and mCHH levels, were maintained throughout seed ripening and germination processes, we asked if the global mCG level difference between both ecotypes is also maintained from local regions or not. First, we calculated the difference in methylation levels of each 50 bp window at each stage and each of the genomic features. Then, we defined eDMR from comparable CR where the DNA sequences exist in both ecotypes as the regions representing between-ecotype Δ(mC level) more than a standard deviation from the global mean of Δ(mC level) at each stage. Overall, CG eDMRs are more strongly maintained than CHG or CHH eDMRs during development (Table 1). Specifically, more than 89% of CG eDMRs (58,923) of a stage are maintained during development (Table 1). CHG eDMRs are slightly less maintained than CHH eDMRs in approximately half of conserved DMRs (22,647) during three developmental stages. Interestingly, genic regions are more robustly maintained than other genomic features in all cytosine contexts (Table 1). More than 90% of CG eDMRs (49,205) are well maintained in the genic regions. By contrast, eDMRs are the least maintained in TEs compared to other genomic features, especially for non-CG sites (Table 1).

**Table 1:** The number of differentially methylated regions (DMRs) during seed ripening and germination

	CG				CHG				CHH			
	R&G	FH	AR	GS	R&G	FH	AR	GS	R&G	FH	AR	GS
Total	58,923	66,565	65,382	65,820	22,647	40,812	38,969	45,516	77,406	134,631	127,451	137,852
Gene	49,205	54,651	54,182	54,319	5933	7775	7366	7592	13,981	20,497	19,341	19,227
Gene&TE	464	558	535	532	452	788	732	810	1760	2740	2673	2750
TE	4212	5234	4883	5193	9988	22,260	21,266	26,700	37,089	73,208	67,778	77,278
IGR	5042	6122	5782	5776	6274	9989	9605	10,414	24,576	38,186	37,659	38,597

The size of a window is 50 bp without overlap. R&G, continuous DMR during seed ripening and germination

To further confirm the highly maintained ecotype difference in the genic regions, the mC of each gbM between both ecotypes at each developmental stage was compared. Then, we sectioned the AR stage into four particular parts (copper metallic: methylated only in Col, pink: methylated only in Cvi, azure blue: middle mC levels in both ecotypes, purple: high mC levels in both ecotypes) to track the mC change of each gene in sections in FH and GS stages. Figure 1f clearly shows the presence of many more hyper mCG genes in Col (X-axis) than Cvi (Y-axis). Furthermore, the position of sections was well maintained for genes even in other developmental stages, meaning that each mCG gbM is strongly maintained. This result supports our previous finding that mCG gbM is static and strongly maintained differentially in the ecotypes throughout development. By contrast, the position and aspect of the sectioned parts from the AR stage in TEs are changed in FH and GS stages (Additional file 1: Fig. S2), suggesting mC of TE regions are dynamically changed.

### Different dynamics of the transcriptome and small RNAome are shown across the stages and ecotypes

To better understand the divergent methylome dynamics between Col and Cvi during seed ripening and germination, we examined the mRNA expression of methylation regulators in three developmental stages. The expression patterns of many RdDM pathway genes involved in Pol IV-dependent siRNA biogenesis and Pol V-mediated de novo methylation were similar in both ecotypes (Additional file 1: Fig. S3). For example, in both ecotypes, DRM2 de novo methyltransferase was highly expressed during seed ripening and germination, speculating that the global dynamics of DNA methylation is similar in both ecotypes is tempting.

**Fig. 2:**
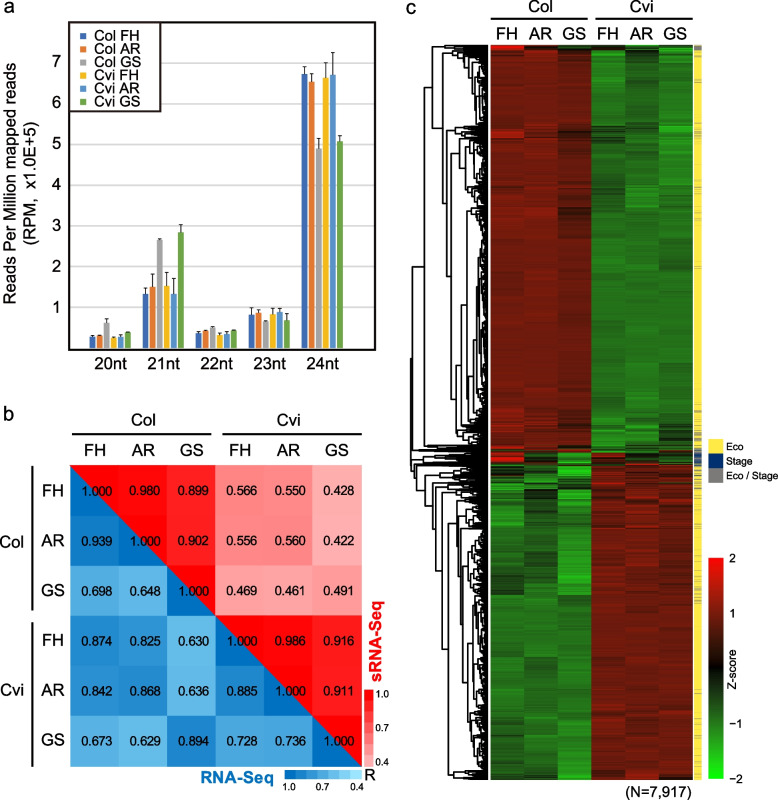
Global Profiling of Transcriptome and Small RNAome from Col and Cvi. **a** Stage-wise small RNA length distribution for Col and Cvi. Reads were counted by reads per million mapped (RPM). **b** Pearson correlation for transcriptome (blue color gradient) and small RNAome (red color gradient) from Col and Cvi. **c** Hierarchical clustering of differentially expressed 24sRCs between stages from Col/Cvi or between the same stage for Col/Cvi. Each 24sRC was defined by ShortStack and their expression levels were measured. Hierarchical clustering was conducted after standardization (Z-score)

To investigate small RNAome dynamics, first, we profiled small RNAs by their length and 5′-end nucleotide. Globally, the length and 5′-end nucleotide distribution of mapped small RNA reads displayed significant compositional changes in 21-nt and 24-nt, which were mainly caused by changes in 21 U (representing AGO1-loaded small RNAs, mainly microRNAs and 21-nt siRNAs) and 24A (representing AGO4-loaded small RNAs, mainly heterochromatic siRNAs) species, respectively, between AR and GS (Fig. 2a, Additional file 1: Fig. S4a). During AR to GS transition, the 20-nt and 21-nt population was increased by 1.8–2.0-fold, and 24-nt decreased by ~ 1.3-fold both in Col and Cvi. To confirm if the decrease of 24-nt small RNA population is genome-wide, we defined clusters of small RNAs using ShortStack and conducted differential expression analysis on 24-nt small RNA clusters (24sRC, *n* = 12,502) between AR and GS was performed (Additional file 1: Fig. S4b, c). We observed 3567 and 97 of 24sRCs showed decreased and increased expression, respectively, during AR to GS transition in Col. Most of these loci changed by 1.2–1.5 fold (FDR < 0.05) (Additional file 1: Fig. S4c), which is similar to the fold-change level of the global 24-nt small RNA population. Only 148 and 62 loci of those 24sRCs were down- and up-regulated more than twofold during the same period (Additional file 1: Fig. S4c). This was similar to what we observed from the fold-change of global 24-nt small RNA population. Together, this suggests that the decrease of the 24-nt small RNA population during AR to GS transition occurs in global rather than limited to specific genomic loci.

To find the difference of the dynamics between transcriptome and small RNAome, correlation and differential expression analysis between stages and ecotypes were performed. Transcriptome and small RNAome profiling across 6 samples showed a significant correlation between FH and AR in Col and Cvi (Fig. 2b). However, differences were found in dynamics between the transcriptome and small RNAome in detail. Compared to the high between-ecotype correlation in the mRNA transcriptome (*Pearson*, R = 0.87–0.89), the small RNAome displayed a more divergent profile between both ecotypes in the same developmental stage (*Pearson*, R = 0.49–0.566) (Fig. 2b). On the contrary, a comparison between GS and the other two stages in small RNAome showed a stronger correlation (*Pearson*, R = 0.89–0.916) than in the transcriptome (*Pearson*, R = 0.64–0.73), which is similar to the level of correlation observed in the comparison between Col GS and Cvi FH/AR and vice versa (*Pearson*, R = 0.63–0.67) (Fig. 2b).

Accordingly, our differential expression analysis reflected the correlation results (FDR < 0.05, fold-change ≥2-fold). A substantial portion of protein-coding genes was differentially expressed from Col and Cvi in a stage-wise manner (10.0 k, ~ 95%) (Additional file 1: Fig. S5a-e), which is mainly comprised of DEGs between AR and GS in both ecotypes (Col: 7161, ~ 90.5%; Cvi: 6359, ~ 76.6%) (Additional file 1: Fig. S5b,c). 35% of the DEGs could explain the inter-ecotype differences (Additional file 1: Fig. S5a). In small RNAome, we confirmed 7917 of 12,502 24sRCs exhibited differential expression from stage-wise and/or ecotype-wise comparison. Among these 7917 24sRCs, 98.9% (7835) were differentially expressed between Col and Cvi in at least one stage. Meanwhile, 8.8% (702) of 24sRCs exhibited differential expression between stages in Col and/or Cvi (FDR < 0.05, fold-change ≥2 fold), and most of them (620 loci) also exhibited ecotype-wise differential expression (Fig. 2c, Additional file 1: Fig. S5f, i, j). These results are consistent with the previous report in maize in that transcriptome are more divergent between roots and shoots while siRNAs are more associated with TEs and repetitive elements [27]. Taken together, our results demonstrate that the global dynamics of transcriptome and small RNAome across the stages and ecotypes act differently.

### Methylation levels on eDMRs are positively correlated with differential expression of 24-nt small RNAs

7835 of 12,502 24sRCs (~ 62.6%) were differentially expressed in ecotype-wise manner (Additional file 1: Fig. S5f). Considering that 24-nt small RNAs play crucial roles in the Pol V-mediated RdDM pathway and one of their major targets is TE [1, 28], ecotype-specific distribution of 24-nt small RNAs might be related to the formation of ecotype DMRs (eDMRs) on TE regions. First, we examined if eDMRs and TE regions are associated with these 24sRCs in an ecotype-dependent manner. To do this, we defined those 7835 of 24sRCs as “e24sRC” (ecotype-wise differentially expressed 24sRC) loci. For consistency in our analysis, we selected 6512 of e24sRC loci exhibiting differential expression between Col and Cvi across all three developmental stages, which were further classified into two groups; e24sRC-Col (3959 loci) exhibiting higher expression level in Col than that in Cvi, and e24sRC-Cvi (2553 loci) exhibiting higher expression level in Cvi than that in Col (Additional file 1: Fig. S5i, j). Similarly, eDMRs that were consistently hyper- or hypo-methylated between ecotypes across stages were chosen for further analysis.

**Fig. 3:**
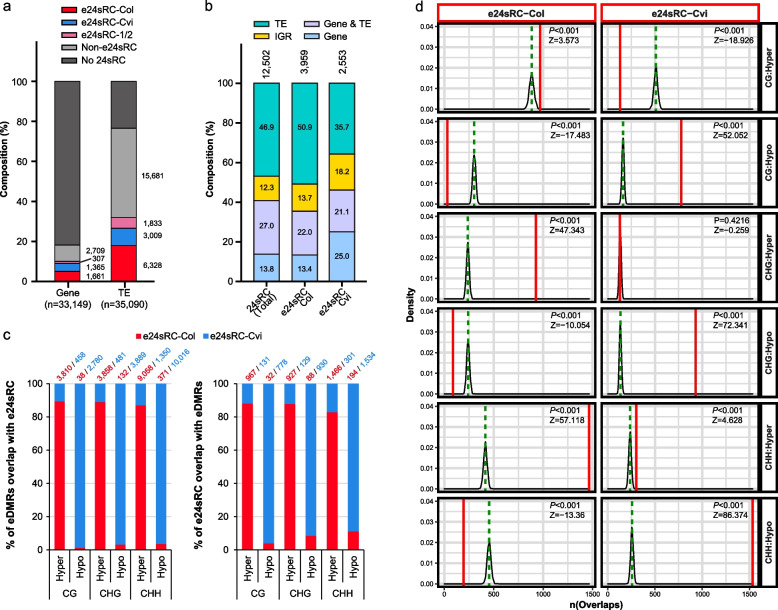
Assessment of 24-nt siRNA clusters (24sRC) enrichment for genomic features and ecotype-specific DMRs (eDMRs). **a** Composition of genes and transposable elements (TE) overlap with e24sRC-Col (red), e24sRC-Cvi (blue), e24sRC-1/2 (pink, 24sRCs exhibiting differential expression between ecotypes in one or two stages) and non-e24sRC (grey). Features that did not overlap with any of the 24sRCs are represented as grey. Numbers near the colored part of the bar graph represent numbers of features that overlap with each category of 24sRCs. **b** Composition of 24sRCs, e24sRC-Col, and e24sRC-Cvi overlap with genomic features, including gene, TE, and intergenic region (IGR). **c** Numbers and compositional ratio of ecotype-specific differentially methylated regions (eDMRs) overlapping e24sRCs (left panel), and that of e24sRCs overlapping eDMRs (right panel). Hyper: hypermethylated region in Col; Hypo: hypermethylated region in Cvi. **d** Randomization-based permutation test results. e24sRC loci (red-boxed) were 1000-times randomly re-distributed across the *Arabidopsis* genome, and to measure the numbers of overlaps between them, each randomly re-distributed feature set was compared with eDMR loci (black-boxed). Green dashed line represents the average distribution for numbers of overlaps between randomly re-distributed e24sRC loci and eDMR loci. Red solid line represents observed overlaps with e24sRC loci and eDMR loci. The X-axis represents the numbers of overlaps between e24sRC loci and eDMR loci, and the Y-axis represents the density value for distribution

To characterize the genomic distribution of e24sRC, we confirmed the number of overlaps of e24sRC loci with genes and TEs from Araport11 annotation [29]. As expected, both e24sRC-Col and e24sRC-Cvi groups showed more significant overlap with TE than the genic region, similar to other 24sRC loci (Fig. 3a). However, clear differences were found between the two groups in the composition of overlapping genomic features (Fig. 3b). TE-overlapping e24sRC-Col loci were more than 50% (reaching ~ 73% if includes “Gene & TE”), while TE-overlapping e24sRC-Cvi comprised only 35.7%, which is even lower than that in total 24sRC. To verify the significance of these overlaps, we conducted randomization-based permutation tests between each group of e24sRC loci and other genomic features analyzed above. Unsurprisingly, the overall e24sRC association with TE or TE-nearby region is significant, and TE was more strongly associated with e24sRC-Col than e24sRC-Cvi, which was expected from the overlap composition results (Additional file 1: Fig. S6a).

Next, we checked the overlaps between e24sRC and eDMRs to see whether methylation level differences are correlated with e24sRCs in a 24-nt small RNA expression level-dependent manner between ecotypes. A considerable number of overlaps were observed between Col-hypermethylated eDMRs (hyper eDMRs) and e24sRC-Col. Accordingly, a similar result was also observed between Col-hypomethylated eDMRs (hypo eDMRs) and e24sRC-Cvi (Fig. 3c). In addition, e24sRC-Col loci significantly overlapped with hyper eDMRs and barely overlapped with hypo eDMRs, and similar patterns were shown between e24sRC-Cvi and eDMRs (Additional file 1: Fig. S6b). The permutation test results between e24sRC and eDMR were consistent with the intersection results; e24sRC-Col loci were strongly associated with hyper eDMRs (Fig. 3d, left panel of “CG:Hyper”, “CHG:Hyper” and “CHH:Hyper”), while e24sRC-Cvi loci were strongly associated with hypo eDMRs (Fig. 3d, right panel of “CG:Hypo”, “CHG:Hypo” and “CHH:Hypo”). Overall results suggested that there are positive correlation between e24sRC expression levels and methylation levels in eDMRs.

Although all three methylation contexts of eDMRs displayed an association with e24sRC loci, CHH-eDMRs showed the strongest association with both e24sRCs, followed by CHG-eDMRs and CG-eDMRs (Fig. 3d). In addition, when we checked how many e24sRC-Col or e24sRC-Cvi loci were overlapped with multiple contexts of eDMRs, we observed that most of CG- and CHG-eDMR were co-exist with CHH-eDMR (Additional file 1: Fig. S6c). Taken together with positive correlation patterns between e24sRC expression levels and methylation on eDMRs, we speculated that differential methylation in eDMRs is mainly affected by RdDM pathway. Since the involvement of RdDM activity can be easily detected at CHH context than CG or CHG contexts in RdDM pathway mutants, we analyzed CHH methylation difference using methylome dataset from *ago4/6/9* and *drm1/2* mutants sampled in mature embryos [14] to address the involvement of RdDM pathway on the formation of eDMR. Results showed that the methylation level of eDMRs along with e24sRCs or Non-e24sRC (fold-change < 2 fold or FDR > 0.05 between ecotypes throughout all developmental stages) were significantly decreased compared to eDMRs that did not overlap with any 24sRCs (Additional file 1: Fig. S6d).

In conclusion, these positive correlations between methylation and 24-nt small RNA expression in eDMRs and the mutant analysis results suggest the contribution of RdDM in regulating methylation levels in an ecotype-specific manner.

### A large composition of TE and intrinsic features in Col-specific regions contribute to hypermethylation

**Fig. 4:**
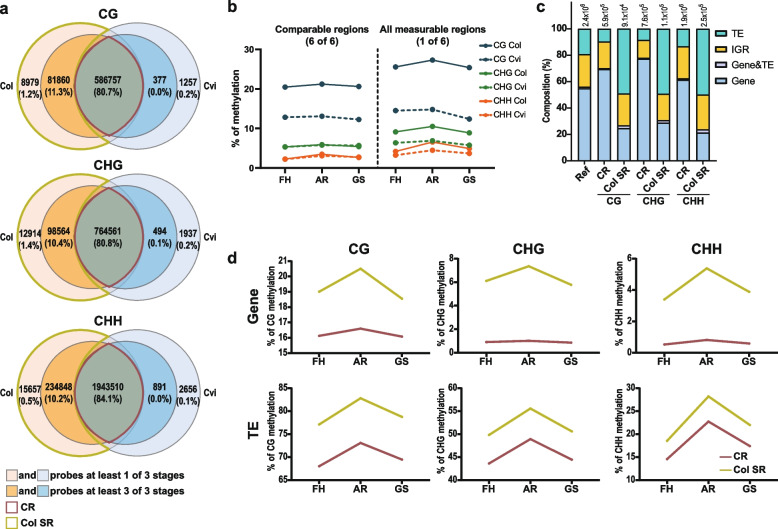
Regions where only the reads of Col were well mapped and its hypermethylation. **a** Venn diagrams for describing how many windows could be analyzed in each ecotype. All numbers indicate the number of 50 bp windows. The windows in the dark red colored intersection (CR) were read in 6 samples and regions, and the windows where only were read enough more than one stage in Col but not in Cvi were colored in lime (Col SR); those two groups were compared in other analysis (**b–d**). **b** Average methylation according to developmental stage in CR (left side) and all measurable regions considered a window in more than one stage. **c** The composition ratio of genomic features in both CR and Col SR was compared. The number of each group is written above each bar. Ref, the composition of TAIR10 genome. **d** Averages of CR and Col SR methylation level of Col according to developmental stages were compared in gene and transposable elements

To compare methylome levels between ecotypes, we selected valid window passing the criteria of 50 bp windows, including at least 3 cytosine contexts with at least 10 reads, in all six samples analyzed. This is represented as an intersection part or common regions called “CR” in Fig. 4a, comprising more than 80% of total windows for each mC context. No significant difference was found in average of non-CG methylation levels between ecotypes during ripening and germination processes in this CR (Fig. 1,4b,b left). However, when we did the same global comparison from all measurable regions, which passed the criteria from at least one out of six samples (Fig. 4a, union for each ecotype), Col exhibited significant higher average non-CG methylation level than Cvi across all three stages (Fig. 4b right). This indicated that the additionally included regions, most of which were comprised of Col-specific regions (hereafter, Col SR) in major, exhibit higher non-CG methylation level compared to CR Because we aligned the sequence reads to the Col reference genome without allowing mismatch, Col SR would contain many sequence differences between the ecotypes. Our result suggests that Col SR is the site that contains higher methylation levels in all cytosine contexts than in comparable intersection regions.

Therefore, we hypothesized the presence of a correlation between DNA sequence diversity and DNA methylation and examined the composition of the genomic features for those ecotype-specific regions. This analysis revealed that Col SR was composed of significantly more TEs for all cytosine contexts (Fig. 4c), which was statistically supported by the permutation test between them (Additional file 3: Dataset S1a). Given that TEs demonstrate generally higher methylation levels than other genomic features, we supposed that the higher mC levels in Col SR were mainly due to the high composition of TE. Interestingly, when we compared the average mC levels of TE from CR and Col SR, we found TE of Col SR shows higher average mC levels than TE of CR (Fig. 4d), which is true for the genic regions as well (Fig. 4d).

In summary, CR displayed very similar levels of non-CG methylation between ecotypes, while relatively diverged Col SR exhibited higher methylation level than CR in Col. The high mC levels in Col SR are not only from a larger TE composition but also from intrinsic properties retained in Col SR.

### The correlation between mC levels and SNPs in Col SR

The possibility exists that Col SR retains many sequence variations, indirectly or directly resulting in higher methylation, and we first considered single nucleotide polymorphism (SNP) as a candidate. However, when we divided Col SR windows into two groups depending on the presence of SNPs or not, the number of windows with SNPs was much lower than those without SNPs in Col SR (Table 2), suggesting other factors beyond SNPs.

**Table 2:** The number of windows including SNP per cytosine context in Col SR

Cytosine context	CG	CHG	CHH
Total No. of windows	90,839 (100%)	111,478 (100%)	250,505 (100%)
No. of windows with SNP	23,232 (25.6%)	25,137 (22.5%)	62,685 (25.0%)
No. of windows without SNP	67,607 (74.4%)	86,341 (77.5%)	187,820 (75.0%)

Single Nucleotide Polymorphism (SNP) was decided by comparing Col and Cvi genome sequence. The size of the windows is 50 bp without overlap. The percentage in the bracket is based on the total number of windows for each cytosine context

**Fig. 5:**
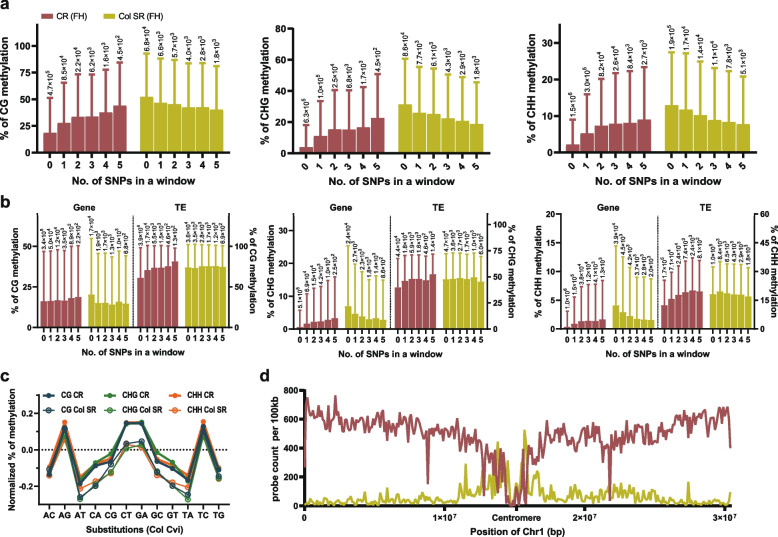
Correlation between SNPs and methylation level and position of CR and Col SR. **a** The correlation between the number of SNPs and DNA methylation is analyzed. Numbers in X-axis represent the number of SNPs in a 50-bp window. **b** The windows overlapped in gene and transposable element (TE) from each CR, and Col SR was analyzed. The positive correlation shown in CR in (**a)** is strongly demonstrated in TE, and the negative correlation shown in Col SR in (**a)** is not shown in the windows overlapped with TE, but it is shown in the windows overlapped with gene. The height of the bar, mean; the length of error bar, std. **c** Methylation level according to each substitution, obtained by comparing Col and Cvi (the first letter is from Col), was normalized with mean and standard deviation ($\frac{x-m}{\sigma }$). **a-c** Methylation level of Col FH was used as a representative. **d** The position of CR and Col SR was analyzed in chromosomal view

Despite fewer SNPs in Col SR, we examined the relationship between the number of SNPs and their DNA methylation levels, if any, in Col SR and in CR for a comparison. To do this, we used methylation levels of the Col FH stage and the SNP information identified by a comparison between Col (TAIR10) and Cvi (Ver.2) reference sequences [30]. Our analysis clearly revealed that Col SR decreased in mC levels, whereas the common CR demonstrated an increase in mC levels as the number of SNP increased, regardless of cytosine contexts (Fig. 5a). If the number of SNPs directly correlates with DNA methylation levels, it would show the same pattern in both regions. Since our analysis showed an anticorrelation of mC with the number of SNPs in Col SR but a positive correlation in CR, only the number of SNPs does not affect mC in Col SR and in CR. These results were reproduced when we used Col AR and Col GS (Additional file 1: Fig. S7).

Next, we tested the correlation between mC and the number of SNPs separately for gene and TE. This revealed that the correlations shown in each group resulted from different genomic features. The positive correlation in CR was contributed not only from TE, but also from gene that comprises at least 60% of the CR genomic features in all cytosine contexts (Fig. 5,4b,c). However, the anticorrelation detected in Col SR is mainly from gene, although its portion in Col SR is less than 30% (Fig. 5, 4b,c). On the contrary, although TE constitutes the most significant genomic portion (approximately 50%) in Col SR, it does not significantly contribute to the anticorrelation between mC levels and the number of SNPs. In conclusion, our analyses reveal that number of SNPs affects mC levels differently on CR and Col SR.

Further, we investigated whether any possible link exists between mC level and a specific substitution. We defined 50 bp windows, including any substitution in CR and Col SR, and then normalized methylation levels with the average and standard deviation. Our analysis revealed that the windows, including one or more of the four transition mutations (AG, CT, GA, and TC), are more methylated than other substitutions (Fig. 5c). Notably, the mC levels of four transition mutations are very similar in CR, but only AG and TC transition mutations are more methylated in Col SR, among other transitions or transversions (Fig. 5c). Therefore, it is tempting to speculate that AG and TC mutations might be either a cause or consequence of the high methylation levels in this region. Alternatively, Col SR might reside in a more heterochromatic region of a chromosome than CR, thereby tending to be more methylated through AG and TC mutations over time. Indeed, we found that CR regions are located throughout the chromosome except in the pericentromeric region, whereas Col SR is located not only in the chromosomal arm but also even more in the pericentromeric region (Fig. 5d).

### Ecotype-specific structural variations in Col SR are hypermethylated

**Fig. 6:**
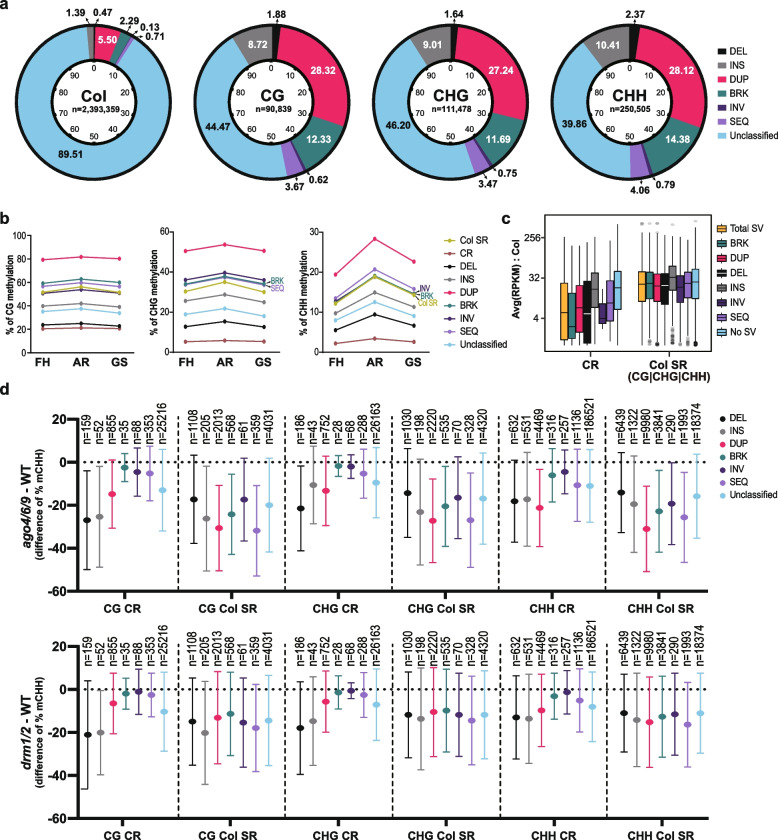
Correlation between structural variants (SVs) and DNA methylation. **a** Structural variants (SVs) were analyzed for Col SR. The proportion of each SV was indicated as %. Unclassified are the regions not included in other SVs. “Col” indicates the composition of Col ref. genome. **b** DNA methylation dynamics of each SV during seed ripening and germination were analyzed. The average level of methylation is represented as dots and linked with a line (**b, d**). **c** Average 24-nt small RNA expression level of Col samples overlaps with CR or Col SR. “Col SR (CG|CHG|CHH)” indicates genomic loci covered by at least one of three types of cytosine methylation context windows in Col SR, and these loci were used to find 24sRCs overlap with Col SR. 24sRCs that did not overlap with “Col SR (CG|CHG|CHH)” loci were treated as CR-overlapping 24sRCs. **d** CHH methylation differences between wild type and RdDM machinery mutants in SV-overlapping CR and Col SR. Sequence-gain type SVs in Col SR were more hypomethylated than those in CR in mutants

Since the number of SNPs does not explain the high mC levels in Col SR, we examined structural variances (hereafter, SVs) within Col SR by comparing TAIR10 and the de novo Cvi genome [30] using a whole-genome sequence comparison tool (MUMmer, see Methods). SVs are categorized and described in an aspect of Col into six groups and the other unclassified SVs (Fig. 6a); a deletion (DEL), an insertion (INS), a duplicated sequence (DUP), a breaking insertion (BRK), an inverted sequence (INV), and a translocation event (SEQ). For Col whole-genome categorization into the 6 groups above, 89% belongs to the Unclassified followed by DUP, comprising 5.5% of the genome (Fig. 6a).

We also validated significance of enriched MUMmer-predicted SVs in Col SR with permutation assay (Additional file 1: Fig. S8a). In addition, to address the significance of our results, we decided to utilize previously reported Arabidopsis SV loci predicted by Jiao et al. [30] and conducted a permutation test as well. In concordance with the permutation test results for an overlap between MUMmer-predicted SVs and CR or Col SR, our permutation test confirmed that, for all mC contexts, Col SR was strongly associated with most types of SVs, while CR was not (Additional file 1: Fig. S8a). Differences were found in association according to the mC context or type of SVs. For example, CHH of Col SR showed the most substantial association level with “DUP” among MUMmer-predicted SV types (Additional file 1: Fig. S8a, Additional file 3: Dataset S2). In addition, Col SR exhibited a strong association with SVs that are related to sequence-gain type (such as BRK or INS) or copy-gain type (such as “Copy-gain”-tagged SVs of “SyRI-predicted SV” in Fig. S8a) in Col (Additional file 1: Fig. S8a). This is consistent with the result that Col SR is the region where Cvi’s WGBS reads were not well aligned.

As expected, CR contains very few structural variances, especially for the regions where SNPs are low (Additional file 1: Fig. S8b). In contrast, distinct structural features such as DUP, BRK, and INS are more enriched in Col SR (Fig. 6a, Additional file 1: Fig. S8b). Interestingly, more structural variants are included in Col SR when no SNP exists, and then, as SNPs increases, only the DUP and Unclassified classes are found (Additional file 1: Fig. S8b).

To confirm that these structural differences could contribute to the high mC levels in Col SR, we checked the methylation levels in SVs. DUP showed strikingly higher mC levels than other variances (Fig. 6b). BRK, SEQ, and INV generally showed high mC levels. Interestingly, INS is in Col SR with the 3rd higher composition among SVs, but its mC level is even lower than the mean of mC levels of Col SR. Notably, the mC levels of SEQ contribute to a high mC level in Col SR, and even its composition ratio is lower than INS (Fig. 6a, b).

Accordingly, we examined whether those methylation differences are associated with 24-nt siRNA expression or not. As a result, the expression levels of 24-nt siRNAs on each SV type overlapping with Col SR were significantly higher than those with CR (Fig. 6c). Therefore, SVs closely linked to 24-nt siRNAs are one of the factors contributing methylation difference between CR and Col SR.

Since the Col SR includes many SVs with high expression of 24-nt siRNAs and accompanying high DNA methylation levels, we speculated RdDM regulation underlying methylation difference in Col SR. Using publicly available *ago4/6/9* and *drm1/2* mutant methylome at mature embryos [14], we confirmed RdDM dependency on Col SR contributing high mCHH levels. These results demonstrated that between CR and Col SR where both regions displayed RdDM-dependent methylation lost for all cytosine contexts by these mutants, Col SR were more affected than CR (Fig. 6d, Additional file 1: Fig. S8c).

Taken together, along with a previous implication on the relationship between sequence variation and differential methylation [19], our analyses concordantly suggest that hypermethylation in Col SR compared to CR results from, at least partially, the sequence and structural differences from Cvi and is regulated by RdDM pathway.

### Methylation of Col SR-enriched genome rearrangement hotspots are contributed via RdDM pathway

We observed that Col SR loci were significantly enriched with SVs (Fig. 6a, Additional file 1: Fig. S8a), suggesting that these loci underwent drastic sequence divergence between Col and Cvi compared to CR. There were recently reported regions called “hotspots of rearrangements” (HOT regions) where multiple Arabidopsis ecotypes have independently evolved diverse haplotypes in an ecotype-specific manner because of their rapid sequence changes [30]. These HOT regions shared similar intrinsic characteristics with Col SR; they included more TE and fewer genes compared to colinear regions (CR in our study), and they exhibited high structural diversity, which mainly caused by tandem duplication.

**Fig. 7:**
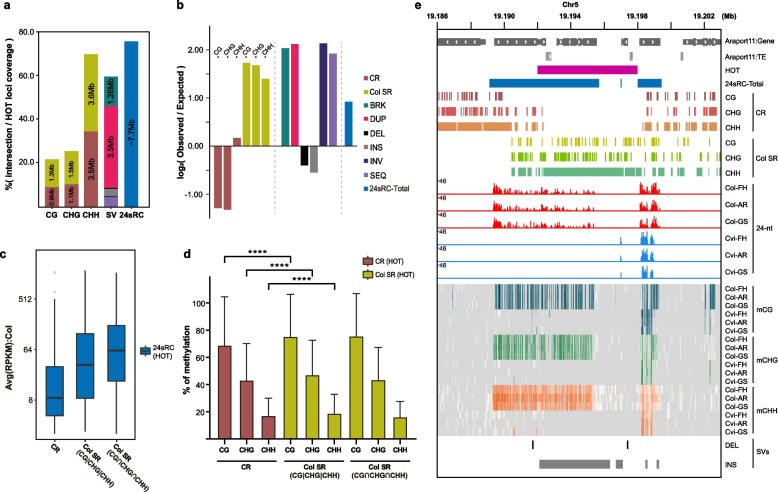
Methylation level in highly sequence-variable HOT regions associated with Col SR, SVs and 24sRC are contributed via RdDM pathway. **a** Ratio and length of features covering HOT region. **b** Bar graphs showing permutation test-derived log_2_(observed/expected) significance for association between HOT regions and each of features tested. Randomization was conducted for merged CR, merged Col SR, each category of SV types, and 24sRC-Total. Permutation was conducted 1000 times (*P* < 0.001). **c,d** Expression levels of 24sRCs **(c)**, and methylation levels in CR and SR **(d)** that are overlapped with HOT regions. “Col SR (CG|CHG|CHH)” indicates genomic loci covered by at least one of three types of cytosine methylation context windows in Col SR, whereas “Col SR (CG∩CHG∩CHH)” indicates loci covered by all three types of cytosine methylation context windows in Col SR. The height of box and the error bar in **d** represent mean and standard deviation, and *P*-value in **d** was calculated with unpaired t-test with *Welch*’s correction. (****: *P* < 0.0001). **e** An example of HOT region covered by 24sRCs and overlapped by Col SR. 24-nt small RNA expression displayed as CP10M-normalized log-scale value. Levels of methylation on each cytosine base displayed as heatmap-style

Because some properties mentioned above were shared between Col SR and HOT regions, we confirmed the overlap between HOT regions and other features analyzed in our study. Among ~ 10.2 Mb of total HOT regions, ~ 55% were overlapped by SVs, mainly by DUP (~ 3.5 Mb) and BRK (~ 1.26 Mb), which is consistent with a previous report that tandem duplication and large indels were shared by many HOT regions [30] (Fig. 7a). We also observed that ~ 4.0 Mb and ~ 3.6 Mb of HOT regions overlapped with Col SR and CR respectively, both of which mainly consisting of CHH context (Fig. 7a), and enrichment significance of CR and Col SR on HOT regions were additionally verified by permutation tests (Fig. 7b). These indicated that those highly sequence-diverse regions between multiple ecotypes were physically associated with loci that we investigated.

We also confirmed that ~ 7.7 Mb (75.6%) of HOT regions were covered by 24sRCs (Fig. 7a). In addition, 24sRCs overlapped with HOT regions in Col SR showed higher expression than 24sRC overlapped with HOT regions in CR (Fig. 7c), which is consistent with differential 24sRC expression observed in SV loci (Fig. 6c). When we checked the methylation level from Col SR and CR overlapped with HOT regions, we observed higher methylation level from HOT region-overlapping Col SR than that in CR (Fig. 7d). Taked together with RdDM mutants analyses results on Col SR (Fig. 6d, Additional file 1: Fig. S8c), these significant coverage of 24sRC on HOT regions and differential methylation between CR- and Col SR-overlapping HOT regions indicated that DNA methylation in HOT regions were also regulated by RdDM pathway suggesting these epigenetic regulation suppresses these rapidly evolving, highly variable sequences between Arabidopsis ecotypes.

## Discussion

Although our transcriptome analysis for RdDM-related genes in Fig. S3 did not provide certain genes responsible for the differences in 24-nt siRNA levels between ecotypes, we discovered regions where the differences in methylation levels between two ecotypes were positively correlated with 24-nt siRNA abundances from significant numbers of eDMR loci. Thus, it is obvious that the differences in methylation levels were originated from the differences in 24-nt siRNA abundances, not from the differences in expression levels of the RdDM-related genes. Since RdDM is well-known for acting in a self-reinforcing manner, it is possible that there are differences in overall efficiency of 24-nt siRNA generation, or differences in copy numbers of 24-nt siRNA-generating sequences between Col and Cvi. Interestingly, Sigman et al. reported recently that 24-nt siRNAs are sufficient to trigger the first round of RdDM by guiding AGO4 and Pol V to loci without pre-existing DNA methylation signatures, and the following self-reinforcing RdDM cycle makes those loci more strongly methylated [12]. Thus, although RdDM pathway gene expressions are the same in two ecotypes, it is tempting to speculate that more copy numbers exist in Col and more efficient 24-nt siRNA generation takes place, resulting in high DNA methylation levels in Col compared to Cvi.

Intriguingly, we found a positive and negative correlation between mC levels and the number of SNPs in CR and Col SR, respectively (Fig. 5). It is tempting to speculate that a selective pressure might be applied in this evolutionary conserved CR, so that as SNPs increase, mC levels also increase, regardless of gene or TE. Given that the amino acid sequences of methylated genes likely evolve more slowly than those of unmethylated genes [31], it is plausible that increasing mC levels help to keep the amino acid sequences unchanged, as SNPs and mutations accumulate during evolution in genes of CR. A recent interesting report suggested that old long terminal repeat (LTR) TEs with an accumulation of mutations over the time could be transcribed again, leading to a secondary RdDM pathway [32]. Thus, mC levels of old TEs in CR may increase to silence TE transcription generating again from the accumulated mutations. By contrast, ‘Col SR’ shows anticorrelation of mC levels with the number of SNP, and this anticorrelation is mainly from gene (Fig. 5a, b). This is interesting because genes in ‘CR’ and ‘Col SR’ show opposite patterns for mC levels to the number of SNP. Specifically, the mC level decrease is striking from SNP 0 to SNP 1 in ‘Col SR’ (Fig. 5b). This result prompts us to think that genes with newly obtained SNPs in ‘Col SR’ lose methylation rapidly, which can alter gene transcription, and this might contribute to a rapid evolution of genes in Col SR, at least in part, compared to genes in CR.

Since HOT regions, as well as SVs, were known to be enriched with TEs and 60% of HOT regions were on pericentromeric region, most of significant overlaps between HOT regions and 24sRCs in Fig. 7a might be due to well-known association between 24-nt small RNAs and TEs. In addition, because TEs in pericentromeric region were known to be hypermethylated via RdDM pathway and caused sequence variations that lead to formation of SVs and ecotype-specific loci like Col SR, high methylation level observed from specific type of SV loci might be a presumable result. However, we observed Col SR locus that were enriched by SVs and HOT regions but barely covered by TEs (Fig. 7e). Like 24-nt small RNA distribution and DNA methylation patterns on RdDM-regulated TEs, these loci showed significant level of 24-nt small RNAs in Col along with all three types of cytosine methylation near or right on Col SR region. Though there were not annotated HOT loci, we found another hypermethylated region enriched with SVs and 24-nt small RNAs without TEs (Additional file 1: Fig. S9). These observations suggested that RdDM pathway contributes to methylation not only on TE-associated SV loci, but also on SV loci that neither were associated with nor were caused by TEs.

## Conclusion

In this study, we conducted multi-omics analysis including methylome, transcriptome, and small RNAome from developing seeds of two *Arabidopsis* ecotypes, Col and Cvi. Through comparing in an ecotype-wise manner, we revealed that ecotype-specific hypermethylation in the common region is positively correlated with ecotype-specific 24-nt small RNA expression, and verified the contribution of RdDM pathway to the formation of those ecotype-dependent DNA methylation patterns in CR. Additionally, we examined the relationship between sequence variation and hypermethylation in Col SR. We found that enriched SVs in Col SR were hypermethylated which seems to be contributed by RdDM pathway during seed ripening and germination in *Arabidopsis*.

In all, our extensive multi-omics study provides insights for understanding the formation and maintenance of ecotype-specific methylation patterns from developing seeds in *Arabidopsis*. These results will help extend our knowledge of the contribution of RdDM-mediated epigenetic regulation on highly variable DNA sequences between Arabidopsis ecotypes.

## Methods and materials

### Plant materials and growth condition

Two accessions of *Arabidopsis thaliana*, Col (Columbia-0, CS22625) and Cvi (Cape Verde Islands, CS1096) used in this study were obtained from ABRC seed stock center. Seeds were sown at MS (Marashige and Skoog) medium, grown until 10 days after germination, and transferred to the soil at 22 °C under long photoperiod (16 hours of light, 8 hours of dark) with cool white fluorescent light (100 μmole/m^2^/s). Fully matured green seeds from brownish siliques just after starting to dry were harvested from both ecotypes and used for FH seeds. Seeds that were harvested from fully dried Col plants were used for Col AR. Cvi seeds harvested from the same staged dried plants were further dried for 60 days and then used for Cvi AR. For GS samples, AR-staged seeds from both ecotypes were treated stratification in the dark at 4 °C for three days, exposed to white light for two hours, and then incubated in the dark at 22 °C for 24 hours.

### Sequencing library construction and data processing

Small RNA-Seq libraries were constructed following in-house protocols [33]. Briefly, small RNAs were selected from the total RNA of each sample by 15% urea-PAGE. Next, eluted size-selected small RNAs were ligated into 3′-adapter and 5′-adapter sequentially, followed by urea-PAGE separation for each step of the adapter ligation reaction. Adapter-ligated small RNAs were amplified using Phusion polymerase (NEB), and then, the amplified products were separated by native-PAGE. Eluted small RNAs were analyzed using HiSeq2500 by Macrogen (Seoul, South Korea). For each stage of seeds from Col and Cvi, three biological replicates of small RNA-Seq dataset were analyzed. Adapter sequence trimming and 18-to-26 nt-length read selection were performed using Trimmomatic (0.39, adapter: TGGAATTCTCGGGTGCCAAGGAACTCCAGTCAC) and Cutadapt (v3.4). Next, trimmed reads were mapped to rRNA/tRNA/snRNA sequences (RNACentral, v17) using bowtie (v1.3.0, −v 1 -m 0 -a) to filter-out structural non-coding RNA reads. All filtered reads were mapped using bowtie (−v 0 -m 0 -k 0). Reads from Col samples were mapped to the TAIR10 reference genome sequence. To align filtered reads from Cvi, the pseudo-Cvi-TAIR10 genome was generated by SNP replacement using Cvi SNP information available in public (ftp://ftp.Arabidopsis.org/home/tair/Sequences/Ecker_Cvi_snps.txt; https://1001genomes.org/data/MPIPZ/MPIPZJiao2020/releases/current).

For generating whole-genome bisulfite sequencing (WGBS) libraries, we harvested samples for multiple times in different days for each stage and each ecotype. Sampling was conducted 5/4/3 and 2/5/2 times for the biological replicates of Col FH/AR/GS and Cvi FH/AR/GS, respectively. We extracted DNAs from each replicate separately. After checking the quality of DNAs, we combined DNAs from each biological replicate to generate a singular library for each stage of each ecotype. This way enabled us to analyze many more regions for the DNA methylation levels with sufficient depth (more than 10x in this study). All of the Whole-genome bisulfite sequencing (WGBS) libraries were constructed using the KAPA library preparation kit (Roche) and EqiTech Bisulfite by Macrogen (Seoul, South Korea). All Sequencing procedure was performed with the HiSeq2000 platform by Macrogen (Seoul, South Korea). 101 bp paired-end reads were generated. All reads were trimmed sequencially by Trim Galore (options, −-clip_R1 10 --three_prime_clip_R1 5) and Trimmomatic (options, SE -threads 16 SLIDINGWINDOW:2:20 MINLEN:40). Then, by using Bismark with bowtie2, WGBS reads of Col and Cvi were mapped to the TAIR10 genome without allowing mismatch. All of the other procedures were described in the previous paper [34].

RNA-Seq libraries were constructed using the TruSeq library preparation kit (Illumina) according to protocols provided by the manufacturer. For each stage of seeds from Col and Cvi, two biological replicates of RNA-Seq dataset were analyzed. Reads from Col samples were mapped to the TAIR10 reference genome sequence, and reads from Cvi sampled were mapped to the SNP-replaced pseudo-Cvi TAIR10 genome using HISAT2 with a non-strand-specific option [35]. Then, to perform reference-based transcript assembly and measure their raw read counts, mapped reads were passed to StringTie [36] and gffcompare [37]. FeatureCounts [38] were then used to measure expression level of genes in TAIR10 and assembled transcripts, and differential gene expression analysis was conducted using edgeR (*glmQLFTest*) [39].

### Calculation of methylation levels and identification of differentially methylated regions (DMRs) and ecotype-specific DMRs (eDMRs)

Factional methylation within 50 bp windows was calculated by the mean level of each cytosine without overlap. We used valid windows including at least 3 cytosines with minimal 10 reads aligned per each cytosine context as a window. For comparing methylation levels between ecotypes and during seed ripening and germination, we used windows valid within all of compared samples. Exceptionally, a 5 kb window including at least 10 sites of cytosine methylation context with more than 10 reads was used for chromosomal view.

In this paper, we defined eDMRs only in common region (CR) by comparing two samples at the same stage with a methylation difference more than or less than a standard deviation from the mean of methylation difference using 50 bp windows satisfying the above conditions.

### Common region (CR) and Col-specific region (Col SR)

In this paper, two groups of regions were defined, common region (CR) and Col-specific region (Col SR), depending on the 100 bp mapping reads from bisulfite sequencing. If a 50 bp window satisfies the conditions of at least 3 cytosines with minimal 10 reads aligned per each cytosine context in both Col and Cvi, the window is included in CR. If a window satisfies the same conditions only in Col but not in Cvi, the window is included in Col SR.

### Methylation level from genebody and transposable element

We used comparable genes and transposable elements (TEs), including at least 5 cytosine sites with a minimum of 10 reads. We sectioned four particular parts from the AR stage; methylated in Col but not Cvi (green), methylated in Cvi but not Col (pink), middle mC level in both ecotypes (cyan), and high mC level in both ecotypes (purple).

### Identification of DNA sequence differences and methylation level of that

For identification of DNA sequence differences between Col (TAIR10) and Cvi (v2.0), we extracted SNPs and structural variations using MUMmer4 (https://github.com/mummer4/mummer). The composition is counted using the number of overlapped windows. According to the number of SNPs, the fractional methylation level (50 bp) was calculated using only Col FH.

### Small RNA clustering analysis and differential expression analysis

To generate small RNA clusters, filtered small RNA reads were mapped to reference genome sequence (Col for TAIR10; Cvi for pseudo-Cvi-TAIR10) with ShortStack -u mode (v3.8.5, mismatch: 0) [40], and then, 18-to-26 nt-length reads from all libraries were clustered together (ShortStack clustering mode, -rpmm 1.0, -pad 75).

To measure small RNA reads between 20 and 24 nt for small RNA clusters and known microRNAs (miRbase, v22), reads were split based on their coordinates and used with each bam file to measure reads. Next, reads were counted for all small RNA clusters using FeatureCounts (RSubread, read2pos = 5, fraction = T, countMultiMappingReads = T) [38]. Then, measured read counts were analyzed by edgeR package to perform normalization and differential expression analysis (*glmQLFtest*) [39]. Small RNA clusters were selected exhibiting FDR (< 0.05) in differential expression analysis. To select significant differentially expressed small RNA clusters such as 24sRC loci in Fig. 2 and e24sRC loci in Fig. 3, fold-change level (≥ 2 fold) cut-off was additionally applied.

### Permutation analysis

Overlap counting between two feature set was mainly conducted with *bedtools intersect* (v2.30.0) with default options. To perform randomization-based permutation test, *overlapPermTest* function in regioneR package (v1.26.1) was used (*n* = 1000, non.overlapping = TRUE, per.chromosome = TRUE, count.once = TRUE) [41]. Query or compared regions, especially DMRs, were merged using *bedtools merge* (−d 1) before using as input dataset not to overestimate significance between compared sets.

## Supplementary Information

**Additional file 1.**

**Additional file 2.**

**Additional file 3.**

## Data Availability

All sequencing dataset of this article can be accessed through the NCBI Bioproject (PRJNA835511). All of statistical results are available in Additional files 2 and Additional file 3.
