# Supplementary material for: Contribution of RdDM to the ecotype-specific differential methylation on conserved as well as highly variable regions between Arabidopsis ecotypes
Source: BMC Genomics. 2023 Jan 20;24:36. doi: 10.1186/s12864-023-09128-4 (PMC9854041; doi:10.1186/s12864-023-09128-4)
Supplement: Supplementary file 1 — Additional file 1. [file 12864_2023_9128_MOESM1_ESM.pdf]

Figure S1

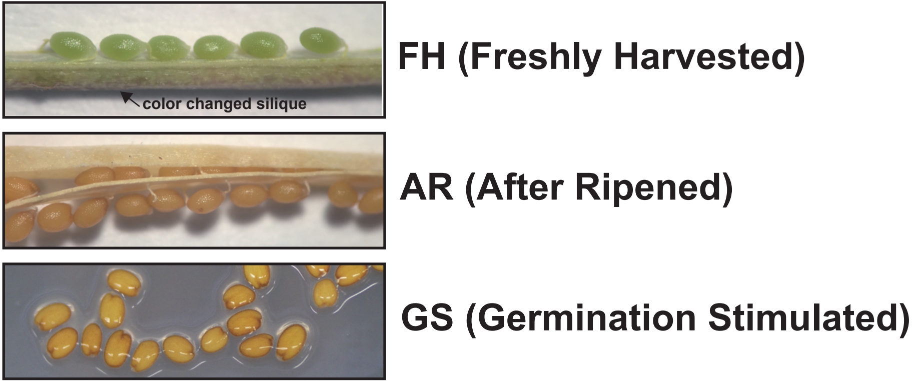
color changed silique
FH (Freshly Harvested)
AR (After Ripened)
GS (Germination Stimulated)

Figure S2

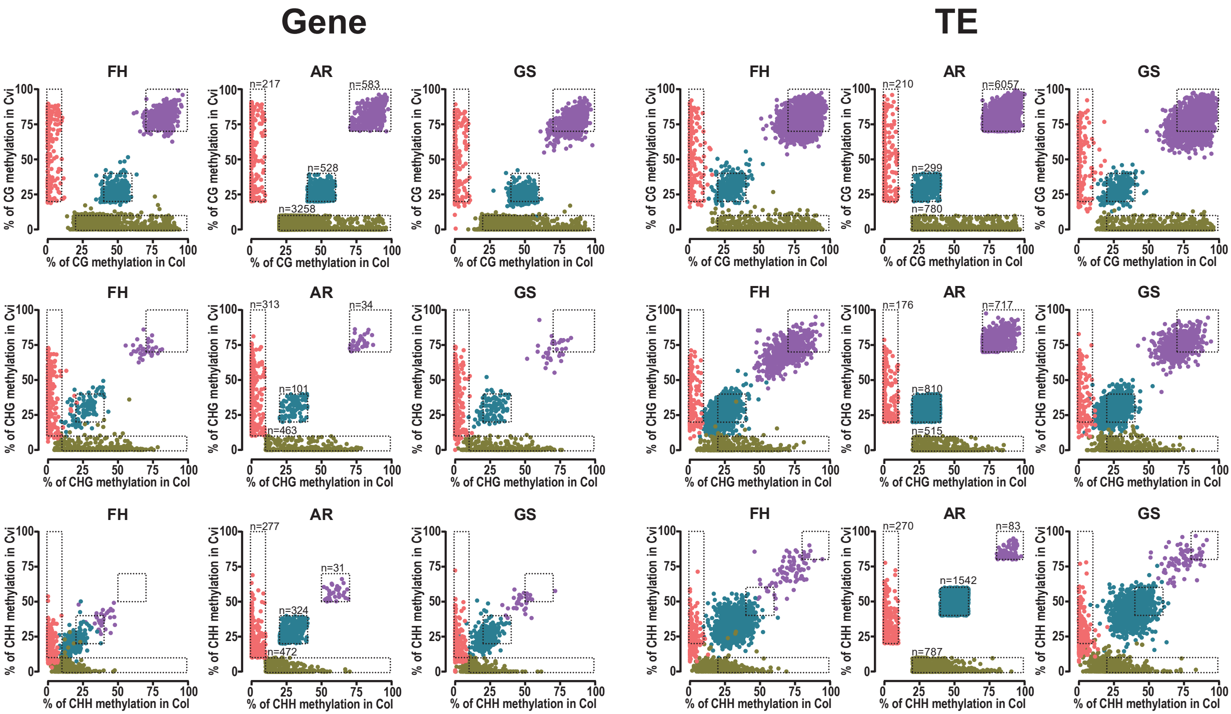
Gene
TE
FH
AR
GS
% of CG methylation in Cvi
% of CG methylation in Col
% of CHG methylation in Cvi
% of CHG methylation in Col
% of CHH methylation in Cvi
% of CHH methylation in Col
n=217
n=583
n=528
n=3258
n=210
n=6057
n=299
n=780
n=313
n=34
n=101
n=463
n=176
n=717
n=810
n=515
n=277
n=31
n=324
n=472
n=270
n=83
n=1542
n=787

Figure S3

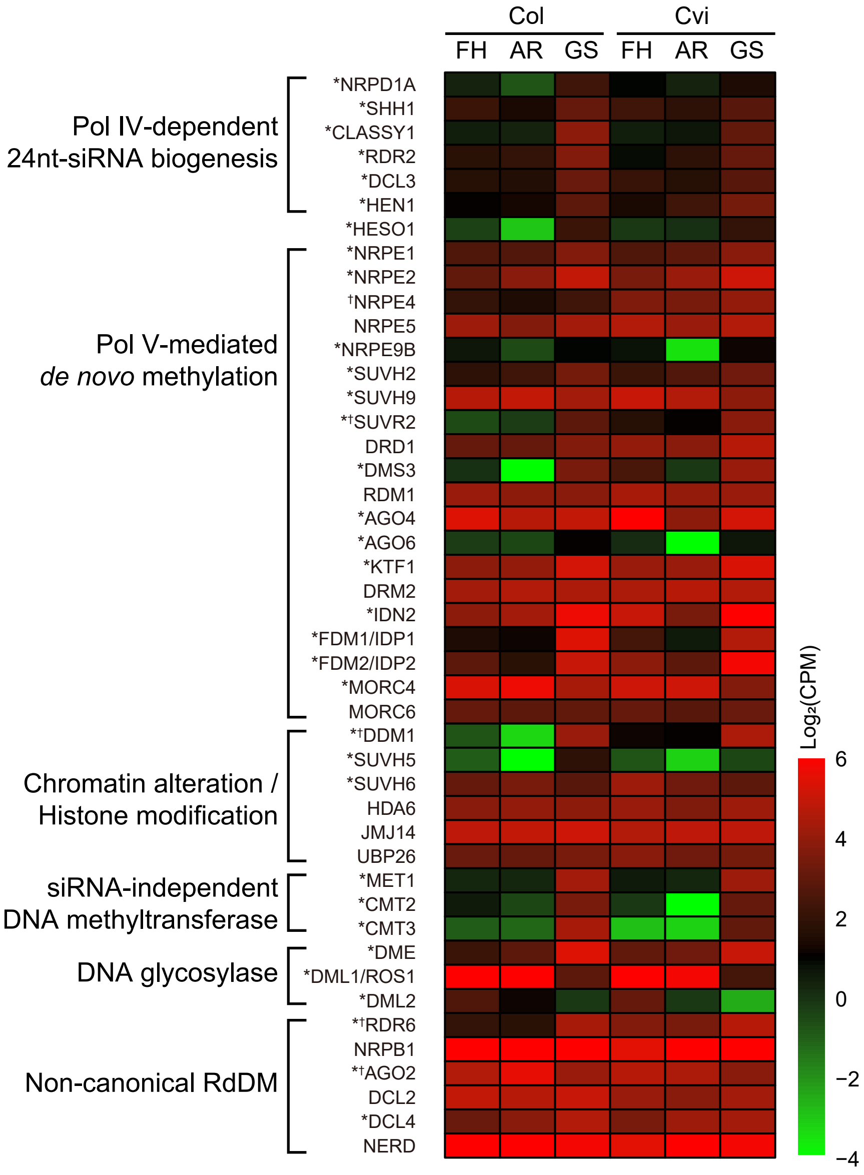

Col
Cvi
FH
AR
GS
FH
AR
GS
Pol IV-dependent 24nt-siRNA biogenesis
*NRPD1A
*SHH1
*CLASSY1
*RDR2
*DCL3
*HEN1
*HESO1
Pol V-mediated *de novo* methylation
*NRPE1
*NRPE2
†NRPE4
NRPE5
*NRPE9B
*SUVH2
*SUVH9
*†SUVR2
DRD1
*DMS3
RDM1
*AGO4
*AGO6
*KTF1
DRM2
*IDN2
*FDM1/IDP1
*FDM2/IDP2
*MORC4
MORC6
Chromatin alteration / Histone modification
*†DDM1
*SUVH5
*SUVH6
HDA6
JMJ14
UBP26
siRNA-independent DNA methyltransferase
*MET1
*CMT2
*CMT3
DNA glycosylase
*DME
*DML1/ROS1
*DML2
Non-canonical RdDM
*†RDR6
NRPB1
*†AGO2
DCL2
*DCL4
NERD
Log2(CPM)
6
4
2
0
−2
−4

Figure S4

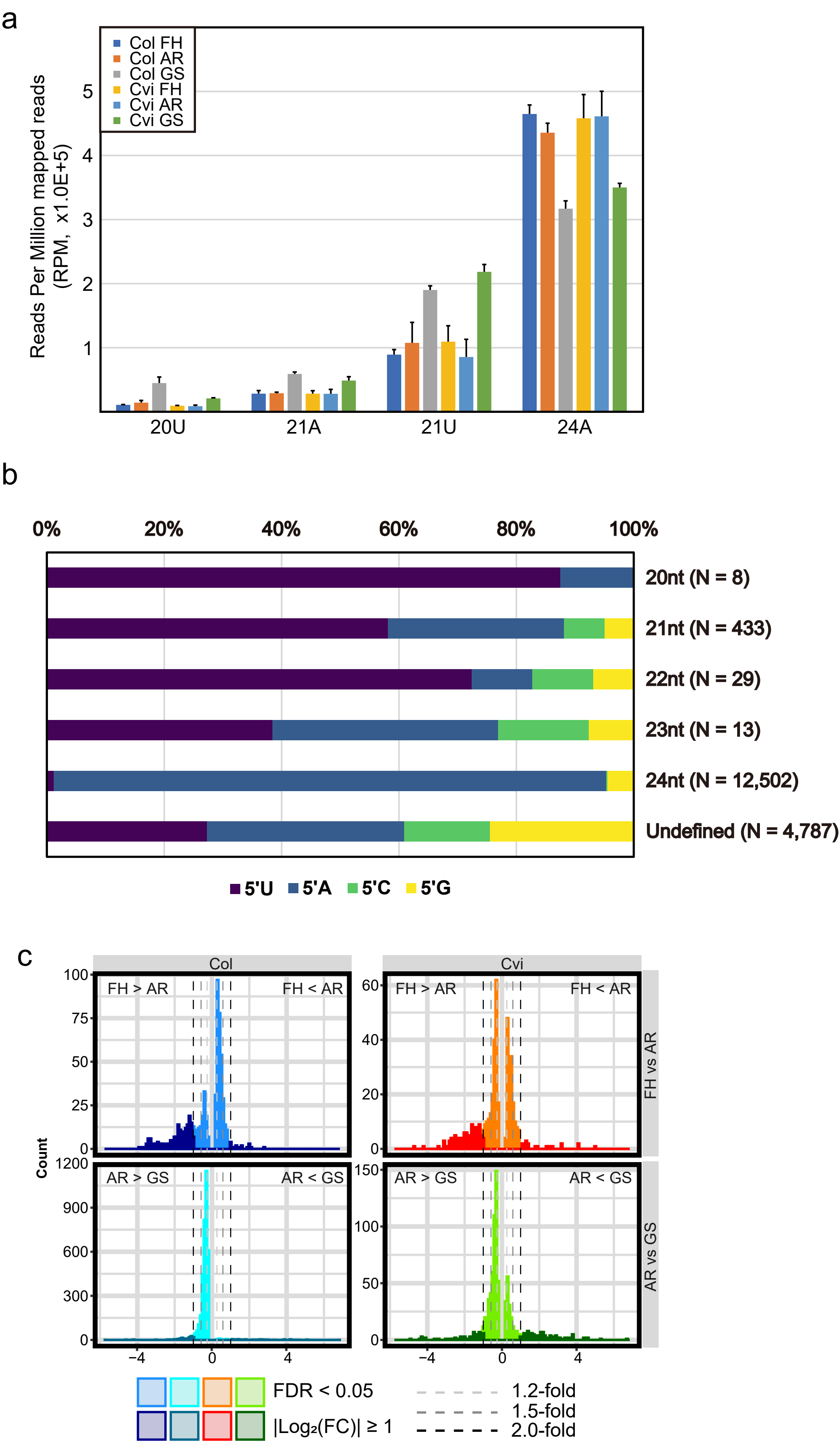
a
Col FH
Col AR
Col GS
Cvi FH
Cvi AR
Cvi GS
Reads Per Million mapped reads (RPM, x1.0E+5)
20U
21A
21U
24A
b
0%
20%
40%
60%
80%
100%
20nt (N = 8)
21nt (N = 433)
22nt (N = 29)
23nt (N = 13)
24nt (N = 12,502)
Undefined (N = 4,787)
5'U
5'A
5'C
5'G
c
Col
Cvi
FH > AR
FH < AR
AR > GS
AR < GS
FH vs AR
AR vs GS
count
FDR < 0.05
|Log2(FC)| ≥ 1
1.2-fold
1.5-fold
2.0-fold

Figure S5

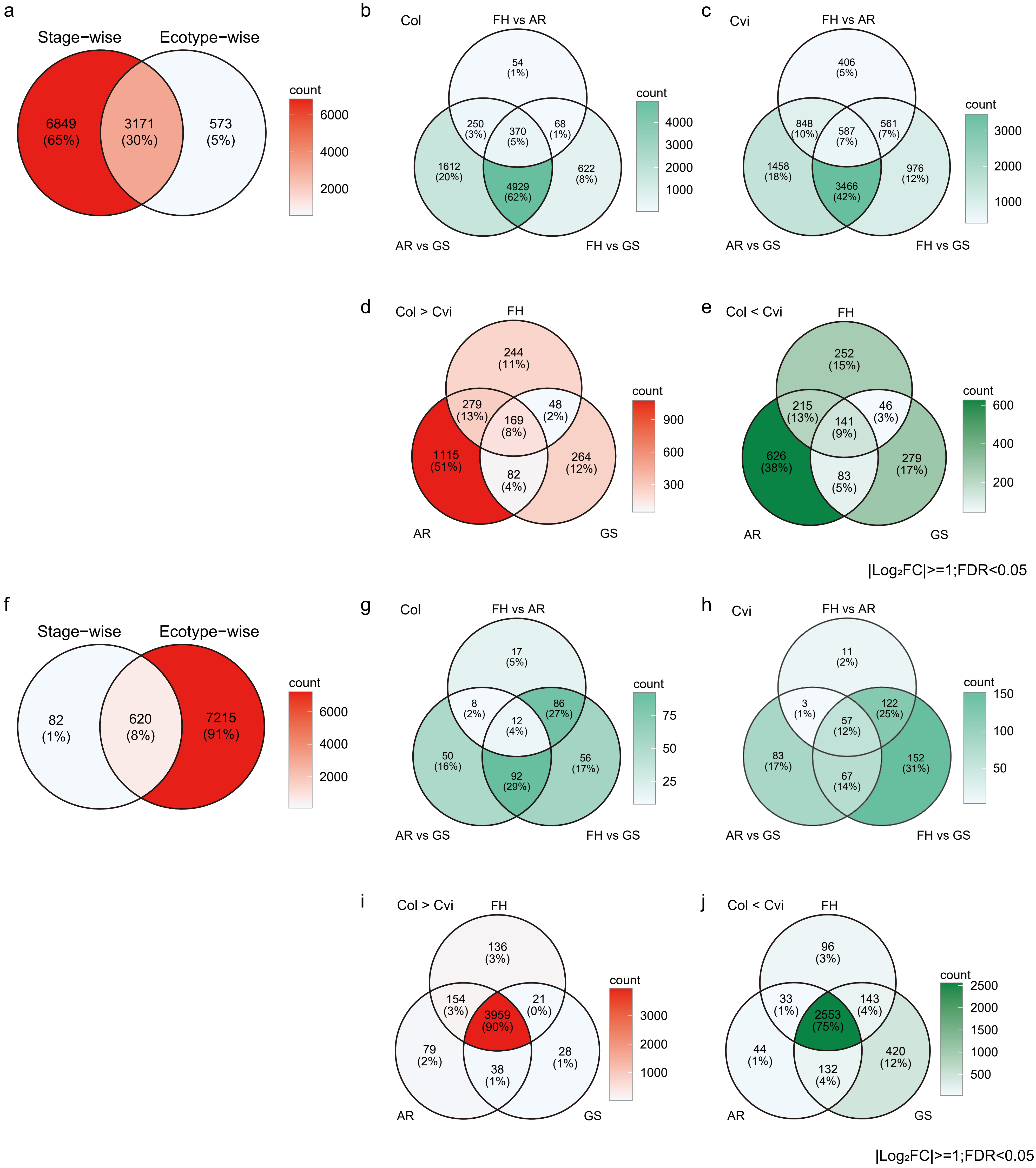
a
Stage−wise
Ecotype−wise
6849
(65%)
3171
(30%)
573
(5%)
count
6000
4000
2000
b
Col
FH vs AR
54
(1%)
250
(3%)
370
(5%)
68
(1%)
1612
(20%)
4929
(62%)
622
(8%)
AR vs GS
FH vs GS
count
4000
3000
2000
1000
c
Cvi
FH vs AR
406
(5%)
848
(10%)
587
(7%)
561
(7%)
1458
(18%)
3466
(42%)
976
(12%)
AR vs GS
FH vs GS
count
3000
2000
1000
d
Col > Cvi
FH
244
(11%)
279
(13%)
169
(8%)
48
(2%)
1115
(51%)
82
(4%)
264
(12%)
AR
GS
count
900
600
300
e
Col < Cvi
FH
252
(15%)
215
(13%)
141
(9%)
46
(3%)
626
(38%)
83
(5%)
279
(17%)
AR
GS
count
600
400
200
|Log₂FC|>=1;FDR<0.05
f
Stage−wise
Ecotype−wise
82
(1%)
620
(8%)
7215
(91%)
count
6000
4000
2000
g
Col
FH vs AR
17
(5%)
8
(2%)
12
(4%)
86
(27%)
50
(16%)
92
(29%)
56
(17%)
AR vs GS
FH vs GS
count
75
50
25
h
Cvi
FH vs AR
11
(2%)
3
(1%)
57
(12%)
122
(25%)
83
(17%)
67
(14%)
152
(31%)
AR vs GS
FH vs GS
count
150
100
50
i
Col > Cvi
FH
136
(3%)
154
(3%)
3959
(90%)
21
(0%)
79
(2%)
38
(1%)
28
(1%)
AR
GS
count
3000
2000
1000
j
Col < Cvi
FH
96
(3%)
33
(1%)
2553
(75%)
143
(4%)
44
(1%)
132
(4%)
420
(12%)
AR
GS
count
2500
2000
1500
1000
500
|Log₂FC|>=1;FDR<0.05

Figure S6

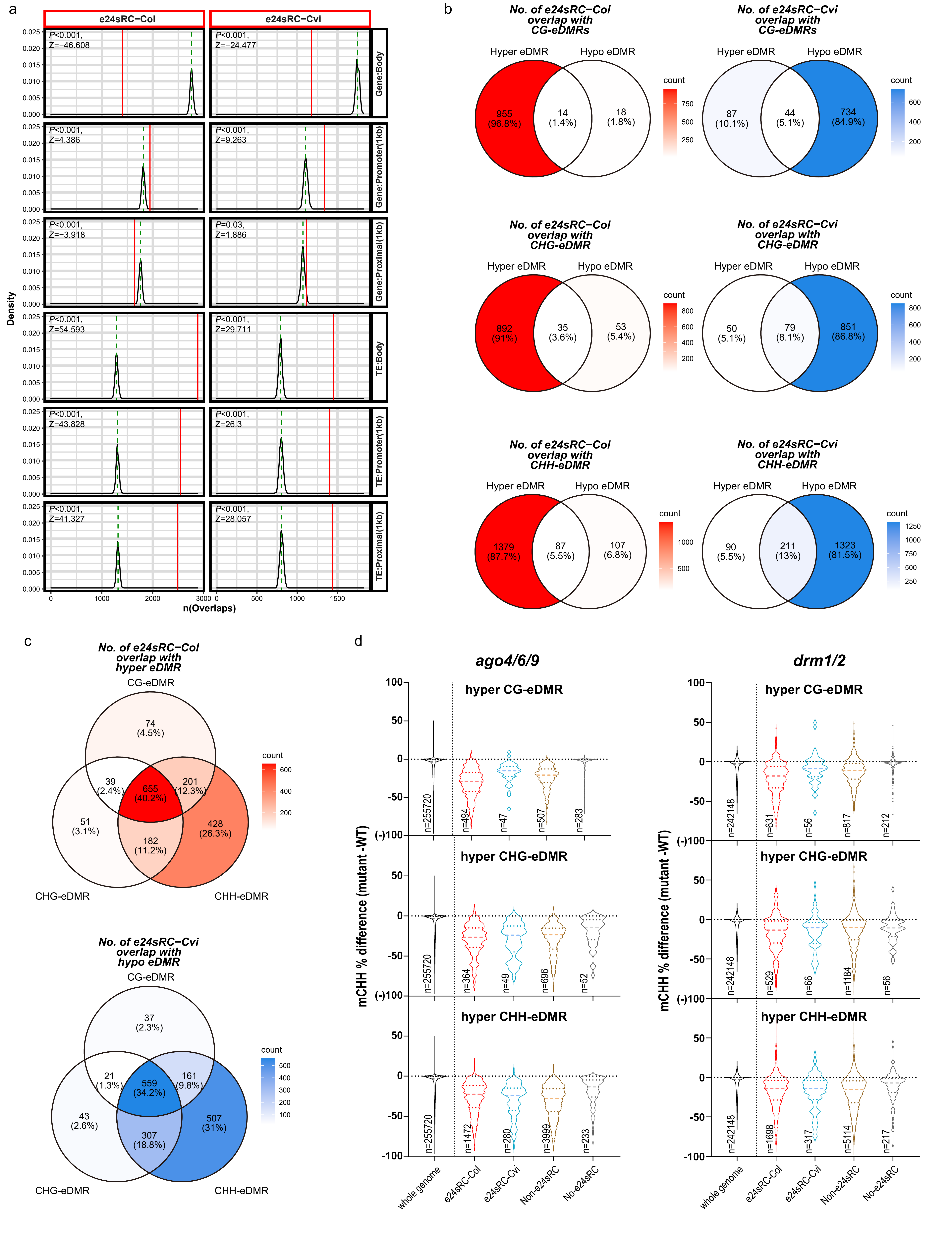
a
e24sRC-Col
e24sRC-Cvi
P<0.001, Z=−46.608
P<0.001, Z=−24.477
Gene:Body
P<0.001, Z=4.386
P<0.001, Z=9.263
Gene:Promoter(1kb)
P<0.001, Z=−3.918
P=0.03, Z=1.886
Gene:Proximal(1kb)
P<0.001, Z=54.593
P<0.001, Z=29.711
TE:Body
P<0.001, Z=43.828
P<0.001, Z=26.3
TE:Promoter(1kb)
P<0.001, Z=41.327
P<0.001, Z=28.057
TE:Proximal(1kb)
Density
n(Overlaps)
b
No. of e24sRC−Col overlap with CG-eDMRs
Hyper eDMR
Hypo eDMR
955 (96.8%)
14 (1.4%)
18 (1.8%)
count
No. of e24sRC−Cvi overlap with CG-eDMRs
87 (10.1%)
44 (5.1%)
734 (84.9%)
No. of e24sRC−Col overlap with CHG-eDMR
892 (91%)
35 (3.6%)
53 (5.4%)
No. of e24sRC−Cvi overlap with CHG-eDMR
50 (5.1%)
79 (8.1%)
851 (86.8%)
No. of e24sRC−Col overlap with CHH-eDMR
1379 (87.7%)
87 (5.5%)
107 (6.8%)
No. of e24sRC−Cvi overlap with CHH-eDMR
90 (5.5%)
211 (13%)
1323 (81.5%)
c
No. of e24sRC−Col overlap with hyper eDMR
CG-eDMR
CHG-eDMR
CHH-eDMR
74 (4.5%)
39 (2.4%)
655 (40.2%)
201 (12.3%)
51 (3.1%)
182 (11.2%)
428 (26.3%)
No. of e24sRC−Cvi overlap with hypo eDMR
37 (2.3%)
21 (1.3%)
559 (34.2%)
161 (9.8%)
43 (2.6%)
307 (18.8%)
507 (31%)
d
ago4/6/9
drm1/2
hyper CG-eDMR
hyper CHG-eDMR
hyper CHH-eDMR
mCHH % difference (mutant -WT)
n=255720
n=494
n=47
n=507
n=283
n=364
n=49
n=696
n=52
n=1472
n=280
n=3999
n=233
n=242148
n=631
n=56
n=817
n=212
n=529
n=66
n=1184
n=56
n=1698
n=317
n=5114
n=217
whole genome
e24sRC-Col
e24sRC-Cvi
Non-e24sRC
No-e24sRC

Figure S7

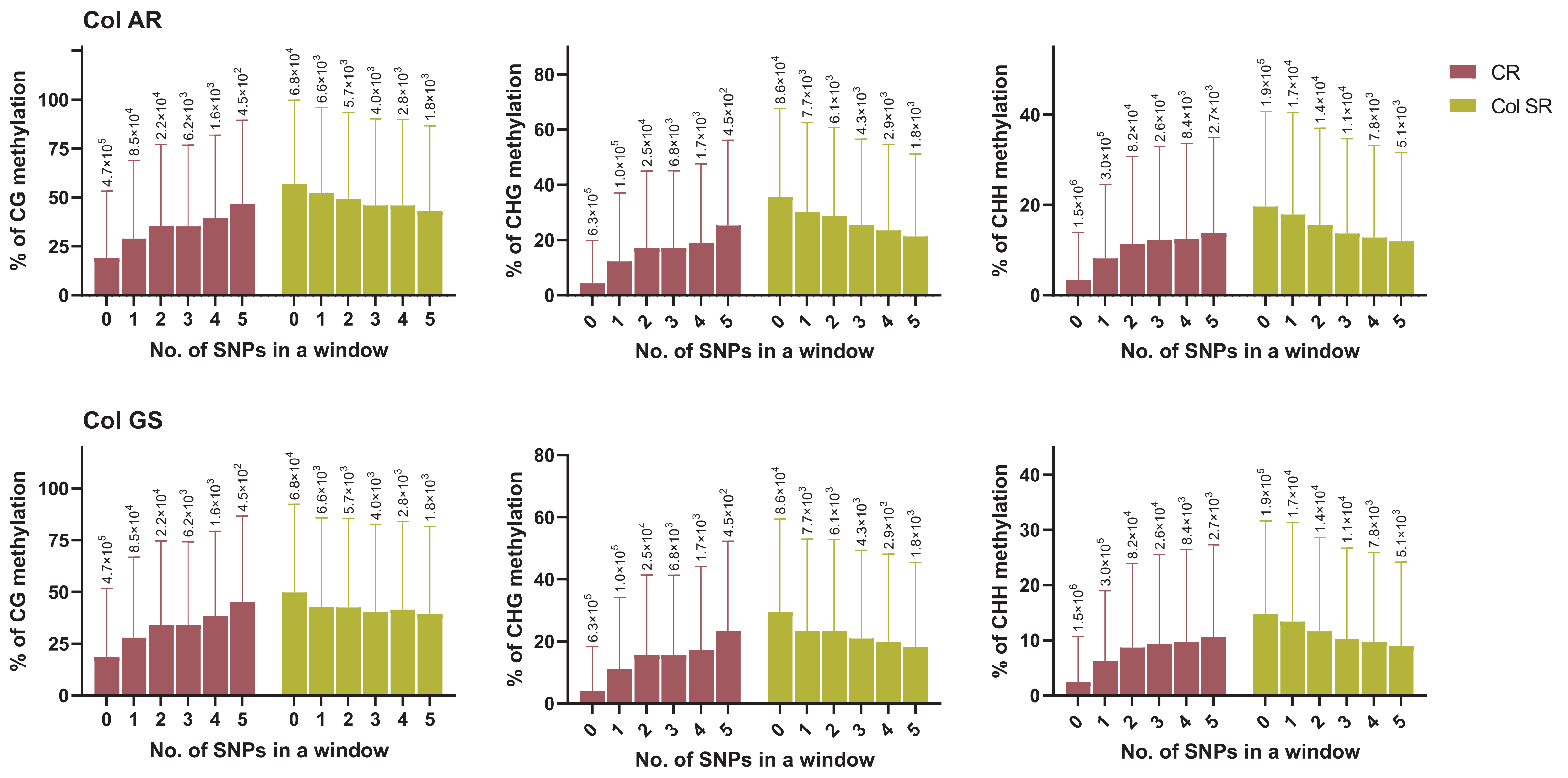
Col AR
Col GS
% of CG methylation
% of CHG methylation
% of CHH methylation
No. of SNPs in a window
CR
Col SR
4.7×10^5
8.5×10^4
2.2×10^4
6.2×10^3
1.6×10^3
4.5×10^2
6.8×10^4
6.6×10^3
5.7×10^3
4.0×10^3
2.8×10^3
1.8×10^3
6.3×10^5
1.0×10^5
2.5×10^4
6.8×10^3
1.7×10^3
8.6×10^4
7.7×10^3
6.1×10^3
4.3×10^3
2.9×10^3
1.5×10^6
3.0×10^5
8.2×10^4
2.6×10^4
8.4×10^3
2.7×10^3
1.9×10^5
1.7×10^4
1.4×10^4
1.1×10^4
7.8×10^3
5.1×10^3

Figure S8

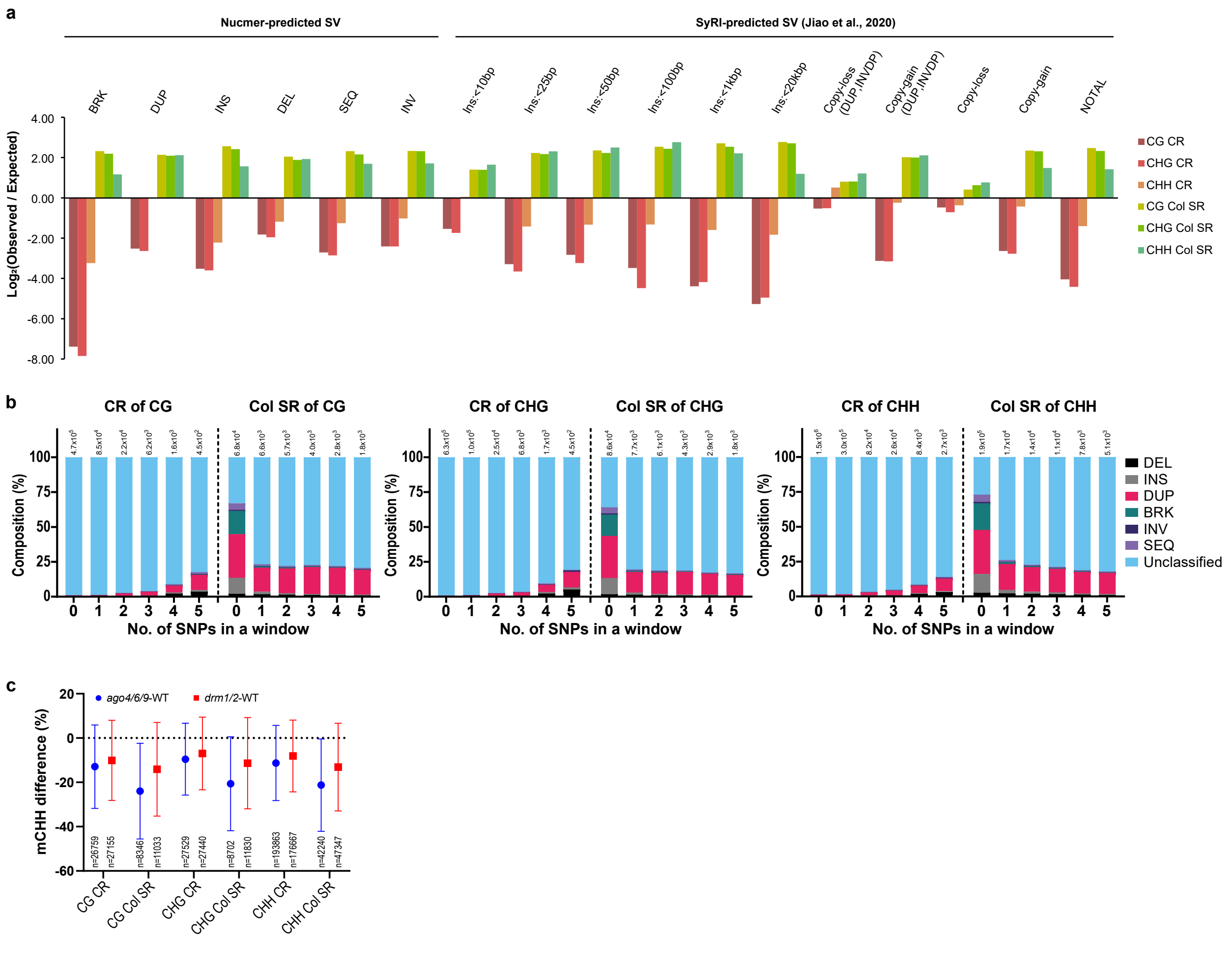

a
Nucmer-predicted SV
SyRI-predicted SV (Jiao et al., 2020)
BRK
DUP
INS
DEL
SEQ
INV
Ins:<10bp
Ins:<25bp
Ins:<50bp
Ins:<100bp
Ins:<1kbp
Ins:<20kbp
Copy-loss (DUP,INVDP)
Copy-gain (DUP,INVDP)
Copy-loss
Copy-gain
NOTAL
Log₂(Observed / Expected)
4.00
2.00
0.00
-2.00
-4.00
-6.00
-8.00
CG CR
CHG CR
CHH CR
CG Col SR
CHG Col SR
CHH Col SR
b
CR of CG
Col SR of CG
4.7×10^5
8.5×10^4
2.2×10^4
6.2×10^3
1.6×10^3
4.5×10^2
6.8×10^4
6.6×10^3
5.7×10^3
4.0×10^3
2.8×10^3
1.8×10^3
CR of CHG
Col SR of CHG
6.3×10^5
1.0×10^5
2.5×10^4
6.8×10^3
1.7×10^3
4.5×10^2
8.6×10^4
7.7×10^3
6.1×10^3
4.3×10^3
2.9×10^3
1.8×10^3
CR of CHH
Col SR of CHH
1.5×10^6
3.0×10^5
8.2×10^4
2.6×10^4
8.4×10^3
2.7×10^3
1.9×10^5
1.7×10^4
1.4×10^4
1.1×10^4
7.8×10^3
5.1×10^3
Composition (%)
100
75
50
25
0
0 1 2 3 4 5 0 1 2 3 4 5
No. of SNPs in a window
DEL
INS
DUP
BRK
INV
SEQ
Unclassified
c
ago4/6/9-WT
drm1/2-WT
mCHH difference (%)
20
0
-20
-40
-60
n=26759
n=27155
n=8346
n=11033
n=27529
n=27440
n=8702
n=11830
n=193863
n=176667
n=42240
n=47347
CG CR
CG Col SR
CHG CR
CHG Col SR
CHH CR
CHH Col SR

Figure S9

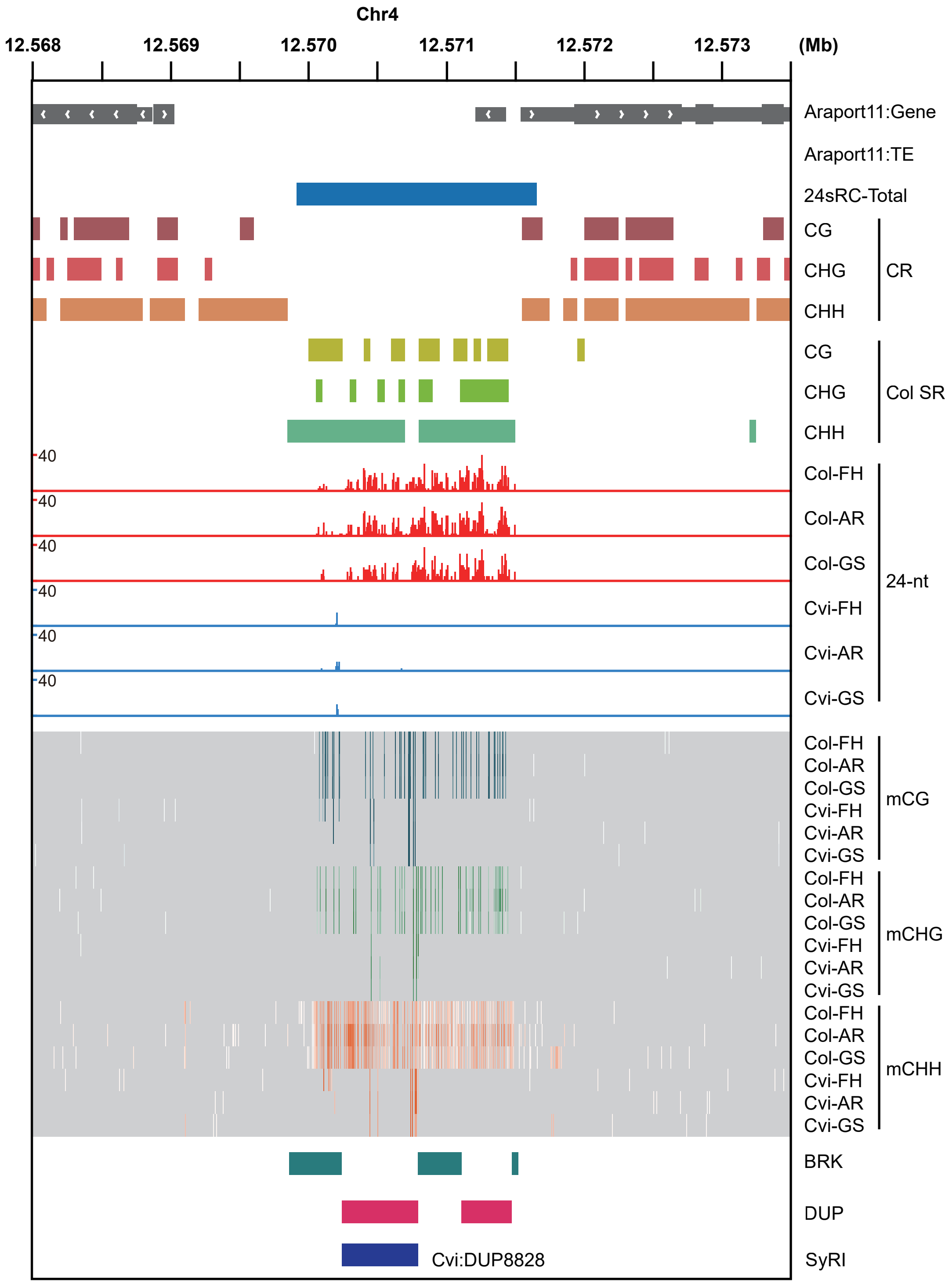

Chr4
12.568
12.569
12.570
12.571
12.572
12.573
(Mb)
Araport11:Gene
Araport11:TE
24sRC-Total
CG
CHG
CHH
CR
CG
CHG
CHH
Col SR
40
40
40
40
40
40
Col-FH
Col-AR
Col-GS
Cvi-FH
Cvi-AR
Cvi-GS
24-nt
Col-FH
Col-AR
Col-GS
Cvi-FH
Cvi-AR
Cvi-GS
mCG
Col-FH
Col-AR
Col-GS
Cvi-FH
Cvi-AR
Cvi-GS
mCHG
Col-FH
Col-AR
Col-GS
Cvi-FH
Cvi-AR
Cvi-GS
mCHH
BRK
DUP
Cvi:DUP8828
SyRI
